# Activation of cerebellar lobules VI-VII during motor imagery but not during motor activation in unilateral cerebellar hypoplasia

**DOI:** 10.1186/2053-8871-1-6

**Published:** 2014-08-06

**Authors:** Christophe Habas, Mario Manto

**Affiliations:** Service de NeuroImagerie, Centre Hospitalier National d’Ophtalmologie des XV-XX, 28, rue de Charenton, Paris, 75012 France; Unité d’Etude du Mouvement, FNRS Neurologie, ULB Erasme, Brussels, Belgium

**Keywords:** Compensation, Neocerebellum, Hypoplasia, Motor imagery, Motor execution, Default-mode network

## Abstract

We report the case of a 25 year-old patient who underwent morphological and functional brain magnetic resonance imaging (fMRI) to investigate a left neocerebellar hypoplasia discovered incidentally. We compared brain activation during overt and covert finger movements, and haptic discrimination. The contralateral cerebellar hemisphere compensated for mental imagery of hand movements and haptic discrimination, but not for motor execution. Moreover, the resting-state functional connectivity did not show compensatory functional coherence between the right cerebellum and cerebral areas connected with the hypoplastic cerebellum. Our case illustrates for the first time that cerebellar compensatory recruitment is an active, specific process related to task complexity and under the control of executive networks.

## Background

The mechanisms of neurological compensation in cases of unilateral cerebellar hypoplasia are completely unknown. Functional neuroimaging allows the characterization of cerebellar and cerebral functional reorganization (neuroplasticity).

## Case presentation

Unilateral cerebellar hypoplasia is a rare cerebellar malformation affecting part of the cerebellum, sparing the other regions of the brain and is consecutive to a prenatal injury (Alkan et al. [1]). Patients may exhibit a slight to moderate cerebellar syndrome unilaterally to the cerebellar disruption, or may be totally asymptomatic. In this latter case, the discovery of the hypoplasia is usually fortuitous.

The mechanisms of neurological compensation in case of unilateral cerebellar hypoplasia are completely unknown. Experimental studies on the mechanisms of recovery of acute cerebellar injuries indicate that preserved cerebellar regions and extra-cerebellar structures such as the somatosensory cortex play a determinant role in the reorganization of central nervous system pathways (Mackel [2]). Motor imagery (mental simulation of an action without execution) is currently proposed as a way to preserve or improve motor function (Saimpont et al. [3]). The rationale is that mental imagery recruits the same neural circuits which are activated during motor action. Whether this is true or not in patients with a prenatal cerebellar lesion is currently undetermined.

We first acquired morphological data to well-characterize a case of unilateral cerebellar hypoplasia discovered incidentally during a CT-scan for orbit trauma. We compared subsequently the patterns of brain activation during (sensori-) motor execution and motor imagery, as well as brain resting-state in this asymptomatic patient.

## Materials and methods

This 25 year-old patient had no history of neurological, psychiatric or cardiovascular disease. The family history was unremarkable. The details of the MRI investigation are given below. The patient gave his informed consent.

### Functional task

Cerebral and cerebellar activations were studied during the following tasks: 1. unilateral, self-paced finger-to-thumb opposition with the right and left hand (motor task), 2. unilateral haptic representation of small chess pieces with the right and left hand (sensorimotor task), 3. unilateral covert finger-to-thumb opposition with the right and left hand (mental imagery task), and 4. at rest (brain resting-state). Each active tasks consisted of 6 alternating 30-seconds phases of rest and stimulation. For brain resting-state, the patient was instructed to keep their eyes closed and try to remain still. The scan lasted 9 min and 10 seconds.

### Acquisition sequences

The MRI study was performed with a whole-body 3 T clinical imager (General Elelectric Healthcare, Milwaukee, USA) using an eight-channel parallel head coil. The following MRI sequences were applied: sagittal T2-FLAIR CUBE, arterial time-of-flight sequence 3D-TOF, axial anatomical 3-D inversion recovery fast spoiled GRASS (IR-FSPGR), diffusion-weighted single-shot spin-echo echo-planar sequence (b = 0 and 1000s/mm^2^) with 50 non-colinear directions for diffusion tensor imaging (DTI), and, for functional imaging, axial T2 *-weighted gradient echo-planar images (EPI) (TE/TR/flip angle/FOV/matrix/Thickness: 30 ms/3000 ms/90°/24x24cm/64x64/4 mm) covering the whole brain (32 axial slices). The same functional sequence was used for the resting-state: 213 volumes were acquired with 4 dummy volumes acquired at the start of the session to allow for steady-state magnetization.

#### Post-processing

*DTI images* were processed with Volume One software which performed a seed-based deterministic streamline fibre-tracking. Seeds were located in: the dorso-lateral upper medulla oblongata, the right dentate nucleus, the basis pontis, the red nuclei and the dorsolateral lower mesencephalon.

*Functional analyses* were carried out using the tools of FMRIB software library (FSL, version 5; Oxford Centre for Functional MRI of the Brain, UK). Data were successively format-converted, slice timing corrected, motion-corrected (MCFLIRT), co-registered with the corresponding anatomical images and with the template brain of the Montreal Neurological Institute (MNI), spatially smoothed with a 5-mm full-width at half-maximum of Gaussian kernel, intensity-normalized and temporal filtered with a high-pass filter. For the box-car time series data during the experimental tasks were determined using the general linear model approach. The temporal derivative of the expected hemodynamic response was also added to compensate for response-delay variations. Functional images were generated by subtraction analysis, comparing the rest and the active condition on a cluster basis (Z = 3, P = 0.05 corrected for multiple comparison. For resting-state, Independent Component Analysis (MELODIC option) (posterior probability threshold: p = 0.5) was applied to preprocessed data in order to compute correlation maps. Only intraparenchymal clusters whose domain frequency was comprised between 0.01 and 0.1 Hz, were taken into account.

## Results

### Anatomical data

**Figure 1:**
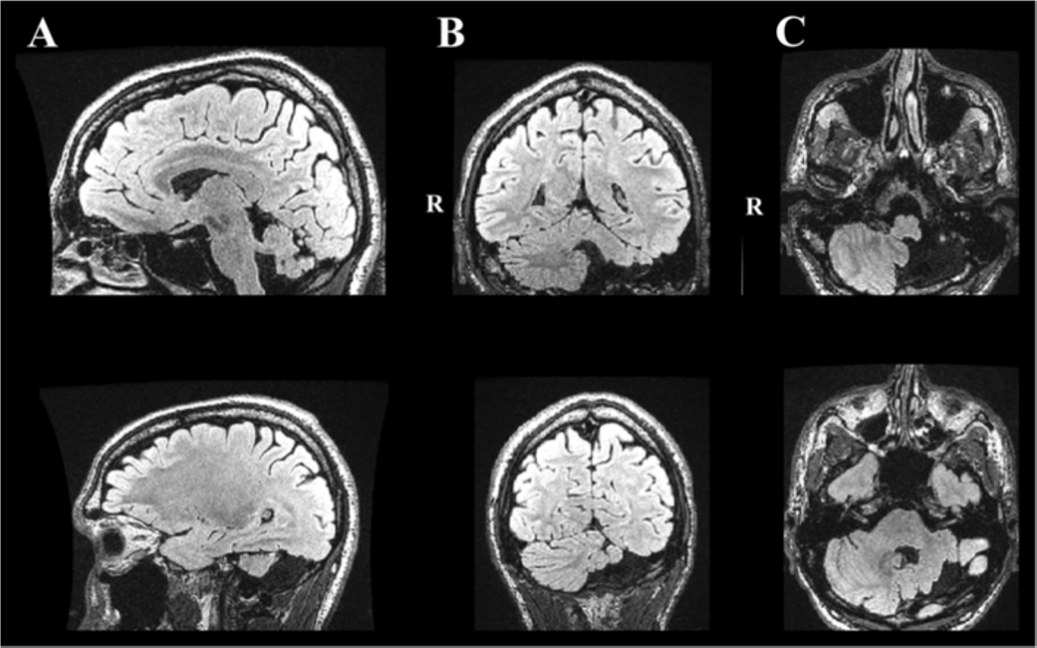
**FLAIR-CUBE image of the encephalon showing left neocerebellar hypoplasia. A**. Sagittal slices. **B**. Coronal slices. **C**. Axial slices passing through the posterior fossa. R. Right side.

**Figure 2:**
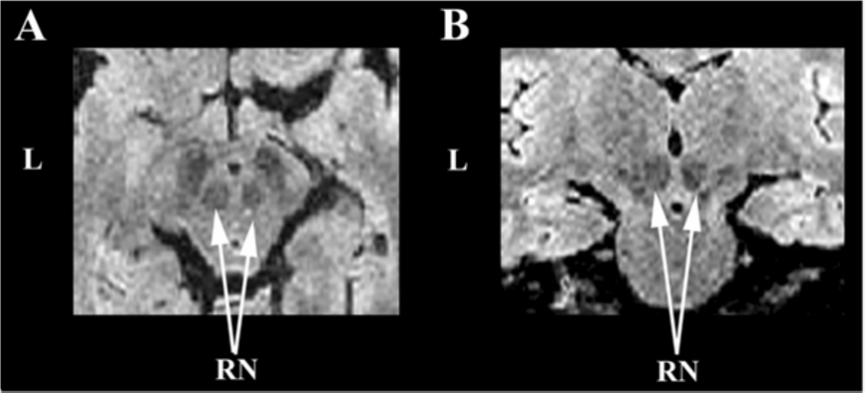
**FLAIR-CUBE image of the red nucleus (RN) whithin the mesencephalon showing a reduced volume of the right nucleus compared to the left one. A**. Axial slice. **B**. Coronal slice. L. Left side.

A left unilateral cerebellar hypoplasia affecting VII/VIII/IX/X lobules and the dentate nucleus was confirmed (Figure 1). We also found a lesser volume of the right red nucleus compared to the left one (Figure 2). On the left side, inferior, middle and superior cerebellar arteries were present but the two former were spindly (not illustrated).

### DTI data

**Figure 3:**
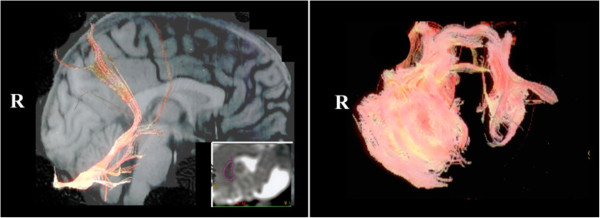
**DTI-based deterministic tractography.** Tract reconstructed using a seed located in the right dentate nucleus and passing through the ipsilateral superior cerebellar peduncle (sagittal slice; image on the left side), and 3D view showing the middle cerebellar peduncles and their expanding within the cerebellar white matter (image on the right side).

DTI showed the absence of the left inferior cerebellar peduncle, the presence of a narrow middle cerebellar peduncle and a vestigial superior cerebellar peduncle on the left side (Figure 3). Therefore, the remaining left anterior lobe could receive afferents from pontine nuclei, but could not send efferents to the diencephalon (thalamus) and brainstem nuclei via the superior cerebellar peduncle, especially to the contralateral red nucleus.

### Functional data

**Figure 4:**
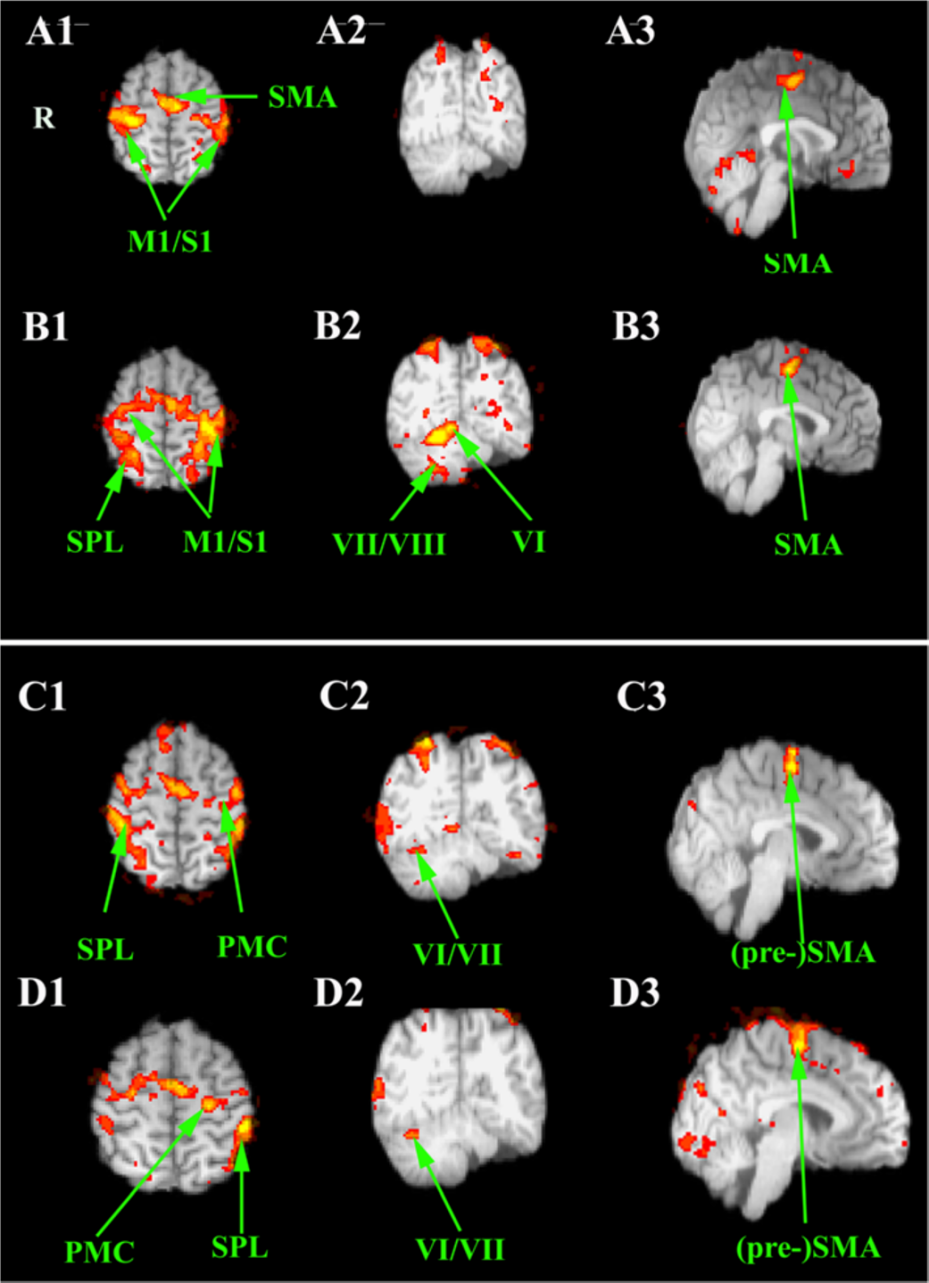
**Statistical parametric maps of the main neural activity of the patient while performing thumb-to-fingers opposition with the left hand (A) and with the right hand (B), and during mental imagery of the same left (C) and right (D) movements.** A1-B1-C1-D1. Axial slices. A2-B2-C2-D2. Coronal slices. A3-B3-C3-D3. No significant cerebellar activation is detected during left movements. Sagittal slices. Abbreviations: ACC anterior cingulate cortex; M1/S1 sensorimotor cortex ; PMC premotor cortex; (pre-) SMA (pre-) supplementray motor cortex; SPL superior parietal lobule. Roman numbers indicate the cerebellar lobules. R right side.

**Figure 5:**
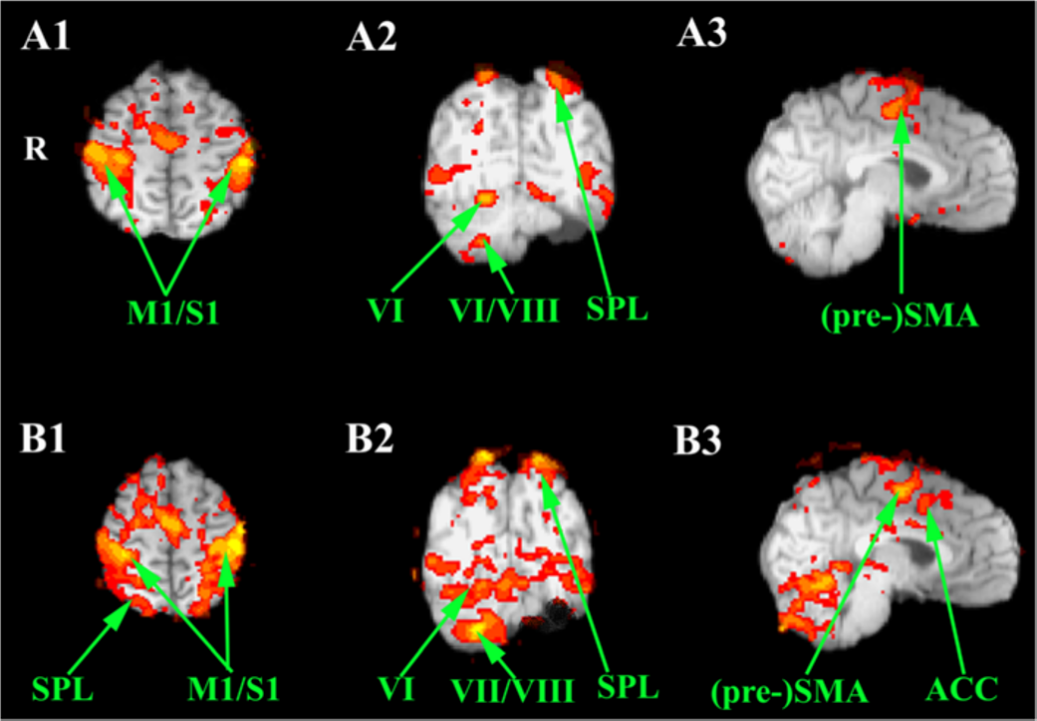
**Statistical parametric maps of the main neural activity of the patient while discriminating haptically small pieces of chess with the left hand (A1-A3) and with the right hand (B1-B3).** A2-B2. Cerebellar activation is detected during movements of both sides suggesting a compensatory role of the right cerebellum during a contralateral sensorimotor task. A1-B1. Axial slices. A2-B2. Coronal slices. A3-B3. Sagittal slices. Abbreviations: ACC anterior cingulate cortex; M1/S1 sensorimotor cortex; PMC premotor cortex; (pre-) SMA (pre-) supplementray motor cortex; SPL superior parietal lobule. Roman numbers indicate the cerebellar lobules. R right side.

In all tasks, we failed to detect any left cerebellar activation (Table 1). For the right hand, we observed an activation of right cerebellar lobules VI and VIII during motor execution (the superior and inferior cerebellar motor homunculi), as anticipated (Figures 4A-4B; Table 1). However, left finger movements did not activate cerebellar lobules VI and VIII. Imagery of both right and left finger movements was associated with a clear activation of right lobules VI-VII (Figures 4C-AD). Moreover, unilateral haptic discrimination with left and right hands also recruited the right neocerebellum (lobules VI-VII-VIII) (Figure 5).

**Table 1:** **Brain areas showing significant activation during the tasks**

Right cerebellar lobules	z-score	x, y, z (mm)*
RH motor task		
VI	11,05	18,-60,-16
VIIb/VIIIa	9,55	22,-66,-48
LH motor task		
VI	4,50	30,-60,-22
VIIb/VIIIa	4,72	32,-64,-52
RH sensorimotor task		
VI	7,18	18,-66,-20
Crus I (/VIII)	7,94	40,-66,-21
LH sensorimotor task		
VI	8,73	22,-64,-18
Crus I (/VIIb)	4,18	40,-70,-24
RH motor mental imagery task		
VI	4,05	34,-54,-24
Crus I	4,11	44,-68,24
LH motor mental imagery		
VI	4,63	30,-62,-20
Crus I	2,55	36,-72,-24

*MNI coordinates ; RH right hand ; LH left hand.

**Figure 6:**
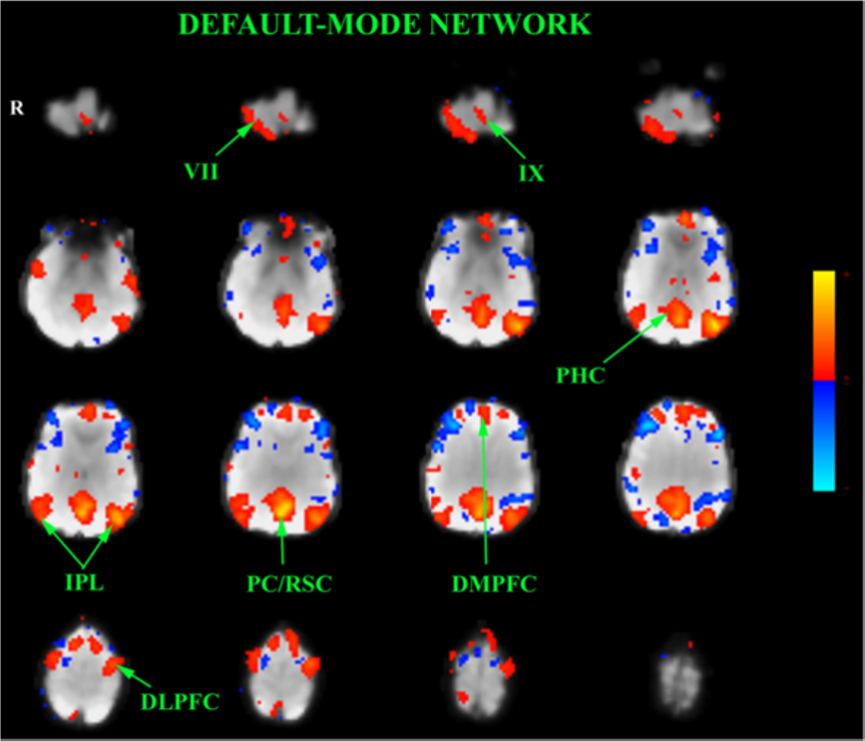
**Spatial map of the independent component computed at rest and corresponding to the default-mode network (red).** Contiguous axial slices of the encephalon from the cerebellum (top and left side) to the cerebral vertex (bottom and right side). Z-score is represented by a color gradient (vertical bar: blue clusters are anticorrelated with the rest-specific red ones). Note that the inferior parietal clusters are less correlated and less extended than the left ones. DLPFC dorsolateral prefrontal cortex; DMPFC dorsomedian prefrontal cortex; IPL inferior parietal lobule; PC/RSC precuneus/retrosplenial cortex; PHC prahippocampus. Roman numbers refer to cerebellar lobules.

Brain resting state showed that: 1. the main canonical intrinsic networks (Habas et al. [4]) were discernable such as: default-mode, right and left executive, visual and sensorimotor networks, 2. the sensorimotor networks displayed no functional coherence with the cerebellum, and 3. the default-mode network was characterized by a decreased and less extended fronto-parietal coherence on the right side compared to the left one (Figure 6).

## Discussion

In this unique case, we demonstrate that the contralateral cerebellar hemisphere compensates for mental imagery of hand movements and haptic discrimination, but not for motor execution. Our case illustrates that the internal representations for motor commands and for mental imagery do not share the same cerebellar circuits in the case of unilateral cerebellar hypoplasia. Our results suggest that an extra-cerebellar zone compensates for hand movements. One possibility would be that the lenticular nucleus could subserve this compensatory mechanism as this structure is involved in early and late motor sequence learning (Lehéricy et al., [5]). However, we did not detect any activation in the striatum although a subthreshold activation cannot be completely ruled out. Alternatively, striatum might have been recruited early in childhood to induce compensatory motor mechanisms in the cereberal cortex for example. However, for complex sensorimotor integration, the contralateral cerebellar hemisphere is still recruited.Therefore, it can be inferred that compensation by the intact cerebellar hemisphere is related to the complexity of the task and, consequently, to the involvement of cognitive systems (attention focusing, working memory, information filtering, decision making, sensory expectations guiding movements). Furthermore, it turns out that this compensation was not reflected in the resting-state functional connectivity mapping since, for instance, no functional coherence was found between the right cerebellum and motor cortical areas, and right executive network. In the DMN, activation of the right fronto-parietal regions interconnected with the left hypoplastic cerebellum was descreased in comparison with the left fronto-parietal ones in relation with the normal cerebellum demonstrating an absence or, at least, a suboptimal compensation by the right cerebellum. One hypothesis might be that cerebellar compensatory action is actively and temporally commanded and controlled by executive networks according to the specificity and the complexity of the task. Passive, spontaneous “mind-wandering”, at rest, should not then require an increased amount of cognitive ressources and, thus, active cortico-cerebellar compensation.

Lastly, given the laterality of cerebellar functions, it can be speculated that the cerebellar hypoplasia could have determined different subclinical or clinical impairments in terms of visuospatial alterations for instance. Alternatively, a right cerebellar hypoplasia could have caused linguistic troubles.

## Conclusion

The attempts to stimulate brain circuits with techniques based on mental imagery should take into account the possible dissociation in the recruitment of networks controlling motor execution on the one hand and motor imagery on the other hand, as reported here. Our patient was clinically asymptomatic, confirming a viable compensatory process. The dissociation observed here could represent a signature of a full recovery process facing a prenatal injury. This should be confirmed in future studies.

## Consent

The patient has given his consent for the case report to be published.
